# Awareness of monkeypox virus among sexual medicine experts is low: a multi-institutional survey

**DOI:** 10.1038/s41443-025-01067-w

**Published:** 2025-04-17

**Authors:** Muhammed A. M. Hammad, Jake A. Miller, Resa G. Magill, Akram Tayser Fattash, Ramazan Omer Yazar, Elia Abou Chawareb, Daniel Sanford, Eliad Amini, Lawrence Jenkins, David W. Barham, Faysal A. Yafi

**Affiliations:** 1 Department of Urology, University of California, Irvine, CA USA; 2 Division of Urologic Surgery, University of Texas Houston, Houston, TX USA; 3 Hospital de Clinicas de Porto Alegre, Porto Alegre, Brazil; 4 Bagcilar Research and Training Hospital, Güngören, Turkey; 5 Department of Urology, University of South California, Los Angeles, CA USA; 6 Department of Urology, University of Tulane, New Orleans, LA USA; 7 Brooke Army Medical Center, Fort Sam Houston, TX USA

**Keywords:** Translational research, Sexual dysfunction, Reproductive signs and symptoms

## Abstract

An international outbreak of Monkeypox (mpox), a zoonotic orthopox virus, was confirmed by the World Health Organization in May 2022. The outbreak represented the first sustained community transmission of mpox beyond West or Central Africa, with speculated causes including declining smallpox vaccination rates, increased international travel, expanding populations, and sexual interactions. This study aimed to assess the understanding and recognition of mpox among sexual medicine experts including the identification of pertained genital lesions. An anonymous electronic survey was developed, addressing clinical manifestations, transmission, and management of mpox. It was distributed to attendees of the 23rd Joint Sexual Medicine Society of North America (SMSNA)/the 23rd International Society for Sexual Medicine (ISSM) conference, 2022. We collected data on various aspects of mpox awareness among the attendees, examining frequencies and percentages of responses. Of 960 conference attendees, 97 (10.1%) responded. Respondents exhibited limited knowledge regarding the recognition of mpox lesions (25.8%), likelihood of anogenital lesions (15.5%), and associated oral or proctitis bleeding (19.6 and 3.1% accuracy respectively). While 78.4% accurately identified contact as the primary transmission mode, knowledge regarding vaccination recommendations (42.3%) and median time from exposure to symptom onset (41.2%) was limited. The survey revealed substantial knowledge gaps among sexual medicine experts regarding mpox. Enhancing education and awareness initiatives is essential to improve preparedness for potential mpox outbreaks, enabling better patient care, and effective management within healthcare systems.

## Introduction

The World Health Organization (WHO) confirmed an international outbreak of the zoonotic orthopox virus, Monkeypox (mpox), in May 2022 [1]. As of December 23, 2022, a total of 83,497 cases of and 72 deaths attributable to mpox have been confirmed by the WHO [1]. The current outbreak represents not only the first occurrence of sustained community transmission of mpox occurring in areas outside of West or Central Africa, but also the first occurrence for which transmission has not been primarily zoonotic [2]. Reasons behind the rapid spread of mpox are speculative, but these may include declining rates of smallpox vaccination, increasing international travel, expanding populations, and increasing rates of sexual interactions [3].

Mpox most commonly presents with a prodromal clinical illness, consisting of fever, headache, back pain, aches, and lymphadenopathy, which typically will last between one to five days [1]. Following this, a characteristic rash will often appear and may desquamate over a period of 2-4 weeks [1]. The characteristic rash associated with mpox involves well-circumscribed lesions with central umbilication and may also include macules, papules, vesicles, pustules, and scabs [4, 5]. Transmission occurs through large respiratory droplets, close or direct contact with skin lesions, and fomites [3]. Some studies have also suggested viral shedding in semen samples as a possible mode of transmission, which may account for the higher rates of transmission seen in men who have sex with men (MSM) and for cases of vertical transmission [6–8]. Secondary bacterial infections involving mpox lesions have been reported and may result in cellulitis of the genital skin and/or balanitis [9]. Anogenital rashes with mpox have reportedly been seen in up to 73% of cases [3, 10]. The varying clinical presentation of mpox can make the recognition of this disease challenging for clinicians, leading to missed diagnoses and delays in treatment [11, 12]. (Table 1).

**Table 1:** Summary of recent monkeypox findings [24].

Category	Details
Major Geographic Regions Affected	• Endemic in Congo, Nigeria • Spread to other African countries • 117 other countries have reported Monkeypox
Prevalence Rates	• Around 95,000 cases were reported as of 4/20/24
Risk Factors	• Healthcare Workers • Individuals with Multiple Sexual Partners
Transmission Modes	• Animal-to-Human Transmission • Aerosol and Droplet Transmission • Human-to-Human Transmission Skin-to-skin Contact Fomites, Clothing • Sexual Transmission • Blood Transmission
Clinical Presentation	• Prodromal Stage • Fever, Malaise, Sweats, Headache • Skin Lesions, typically from genital region • Mucosal Involvement associated with Intestinal Symptoms • Other features include proctitis, phimosis, epiglottis, etc. • Concomitant infection is common
Treatment Options	• Wound Care • Antiviral Medications Potential therapies include tecovirimat, birincidofovir, cidofovir, and VIGIV • No treatments have been validated per FDA
Prevention Strategies	• At-risk populations should consider vaccination • Vaccinations may be given pre or post exposure • Proper PPE for healthcare workers

Given the rates at which mpox may present as a genitourinary rash, and the importance of early diagnosis for the prevention of further transmission, it is imperative that urologists and sexual health experts become familiar with the diagnosis and management of mpox. In this study, the understanding and recognition of mpox by these professionals were assessed.

## Methods

To investigate the presentation, transmission, and management of mpox, an anonymous electronic survey was developed and approved by an institutional review board (IRB) at the University of California, Irvine (IRB# 2220). The survey questions were designed to collect data regarding various awareness details of mpox, including its clinical manifestations, potential modes of transmission, and current management practices. Moreover, respondents were asked to select a picture of a genital rash caused by mpox from a series of pictures that included other differential genitourinary rashes including those of syphilis, lymphogranuloma venereum, molluscum contagiosum. The survey was distributed electronically to a target audience consisting of sexual medicine experts attending the 23rd Joint Sexual Medicine Society of North America (SMSNA) and the 23rd International Society for Sexual Medicine (ISSM) conference, a prominent event in the field of sexual medicine during 2022. After providing informed consent, all participants were reminded that their participation was voluntary and that all responses would remain anonymous and confidential. All data collected were securely stored with access limited to the data analysis team. Survey responses were analyzed using descriptive statistics, namely frequencies and percentages.

## Results

**Fig. 1:**
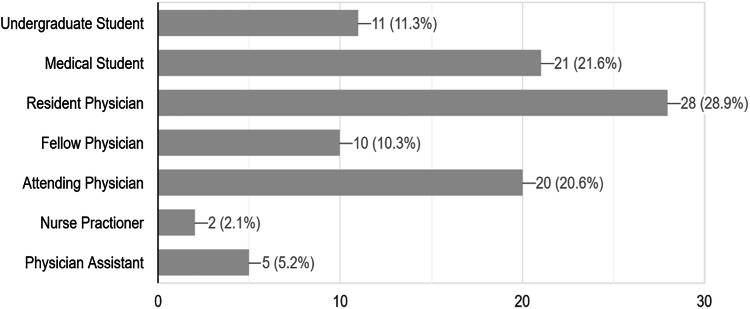
Current Occupational/Trainee Status for the 97 Respondents of the Survey.

**Fig. 2:**
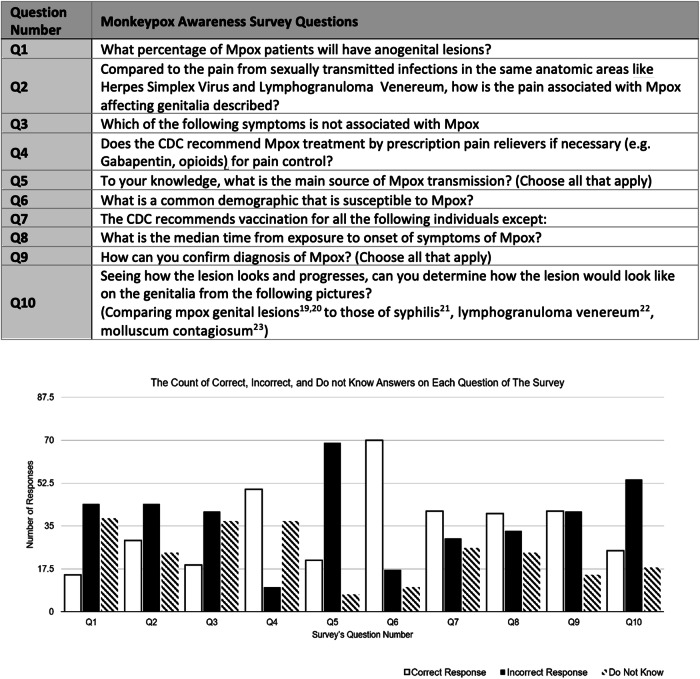
Questions’ table and corresponding graphical representation of the responses & The Count of Correct, Incorrect, and Do Not Know Answer on Each Question of the Survey [19–23].

Of the 960 attendees at the SMSNA/ISSM joint conference, 97 (10.1%) responded to the survey. Resident physicians, medical students, and attendings (28.9%, 21.6%, 20.6%, respectively) comprised the majority of respondents (Fig. 1). The rate of respondents who correctly identified a mpox lesion from a series of cutaneous lesions related to sexually transmitted illnesses (STI’s) was 25.8% (25/97). A similarly low number of 15.5% respondents were able to identify the likelihood of anogenital lesions as mpox presentation. 19.6 and 3.1% respectively knew of the likelihood of mpox patients to develop oral bleeding and proctitis with bleeding on presentation (Fig. 2).

Most participants were able to identify that contact was the primary mode of transmission for mpox, at 78.4% accuracy, and able to identify that homosexual multiple partners were at increased risk of transmission, at 72.2% accuracy. Similarly, most participants (79.4%) were accurately able to identify PCR testing as the preferred choice in confirming mpox infection. However, fewer participants were aware of the CDC vaccination recommendations for mpox, at 42.3% accuracy, or the median time from exposure of mpox to symptom onset, at 41.2% accuracy (Fig. 2).

## Discussion

Given the widespread and rapid transmission of mpox, it is vital that pertained providers remain informed to prevent or limit future outbreaks. The results from our survey indicate a significant knowledge gap amongst sexual medicine experts regarding the presentation, transmission, diagnosis, treatment, vaccination, and clinical recognition of the cutaneous lesions of mpox. All but one question yielded less than 50% correct answers.

Although mpox can have a diverse presentation, responders demonstrated a low knowledge of the typical mpox presentation. Less than 20% of respondents were able to recognize mpox lesions from other sexually transmitted infections. In the prodromal phase, mpox may present only with lymphedema, scrotal pain and a single, painless lesion [10]. As a result, mpox may be difficult to distinguish from certain sexually transmitted diseases, such as secondary syphilis and lymphogranuloma venereum (LV) caused by *Chlamydia trachomatis* [13]. Secondary syphilis can similarly present with systemic symptoms, genital lymphadenopathy, and a rash [13]. However, whereas 36% of individuals with mpox complained of rectal pain and/or pain on defecation, these symptoms are rarely described for syphillis [9]. LV may cause scrotal swelling and pain, but will not present as disseminated and persistent skin lesions [13].

Respondents similarly were inaccurate in regard to the diagnosis and treatment of mpox. Currently, PCR is the modality of choice for laboratory tests, and can detect the virus from the lesion exudate or scabs [14]. The current treatment of mpox, beyond supportive care with symptom control, is stratified by severity of illness and associated comorbidities [15]. The interim CDC mpox treatment guidelines state that antiviral treatment with Tecovirimat (TPOXX) should be initiated in individuals with severe disease (hemorrhage, sepsis, encephalitis), immunocompromised states, pediatric patients, those with exfoliative skin conditions (eczema), woman who are pregnant or breastfeeding, those with multiple complications, and in individuals with involvement of anatomic areas which may result in serious scarring or strictures [15,16].

There are several cases of patients with prolonged penoscrotal edema undergoing successful excision and reconstructive surgery of the tissue, and achieving satisfactory reshaping and sexual function [17, 18]. Other cases have reported surgical intervention for abscess drainage and penile exploration, in a situation when a purulent lesion was identified on imaging [5].

Multiple limitations exist within the current study. Primarily, the survey was self-reported and included a low response rate of 10.1%, which might be explained by the few days of the conference. As such, the survey may be exposed to sampling and non-response bias. Additionally, as the survey was distributed at a conference focusing on sexual medicine, the above results may also not be applicable to the general population of urologists and urology trainees, but rather those interested in or practicing andrology. Future survey studies may be necessary to confirm the above findings and to completely assess the degree to which urologists are comfortable with the diagnosis and management of mpox. Meanwhile, our findings imply that further familiarity and instruction on mpox genital lesions and general information are necessary, especially among pertained subspeciality societies.

## Conclusion

While sexual medicine experts were comfortable with knowledge regarding the transmission of mpox, knowledge on the clinical presentation and management of mpox was lacking. These findings highlight the importance of ongoing education and awareness initiatives to enhance healthcare professionals’ preparedness for mpox cases should future outbreaks occur. By addressing these knowledge gaps, we may improve patient care and contribute to the effective management of mpox within healthcare systems.

## Data Availability

The data that support the findings of this study are available on request from the corresponding author.
